# Development of a PCR assay and pyrosequencing for identification of important human fish-borne trematodes and its potential use for detection in fecal specimens

**DOI:** 10.1186/1756-3305-7-88

**Published:** 2014-03-03

**Authors:** Chairat Tantrawatpan, Pewpan M Intapan, Tongjit Thanchomnang, Oranuch Sanpool, Penchom Janwan, Viraphong Lulitanond, Lakkhana Sadaow, Wanchai Maleewong

**Affiliations:** 1 Research and Diagnostic Center for Emerging Infectious Diseases, Khon Kaen University, Khon Kaen 40002, Thailand; 2 Division of Cell Biology, Department of Preclinical Sciences, Thammasat University, Rangsit Campus, Pathum Thani 12120, Thailand; 3 Department of Parasitology, Faculty of Medicine, Khon Kaen University, Khon Kaen 40002, Thailand; 4 Faculty of Medicine, Mahasarakham University, Mahasarakham 44150, Thailand; 5 Department of Microbiology, Faculty of Medicine, Khon Kaen University, Khon Kaen 40002, Thailand

**Keywords:** Pyrosequencing, Identification, *Opisthorchis viverrini*, *Clonorchis sinensis*, *Haplorchis taichui*, *Haplorchis pumilio*, *Stellantchasmus falcatus*

## Abstract

**Background:**

Small liver and minute intestinal flukes are highly prevalent in Southeast Asia. Definitive diagnosis of parasite infection is usually achieved parasitologically by finding the fluke eggs in feces. However, their eggs are difficult to differentiate morphologically in fecal samples, even for experienced technicians. The present study developed a PCR assay coupled with DNA pyrosequencing for identification of the fish-borne trematodes (FBT), *Opisthorchis viverrini*, *Clonorchis sinensis*, *Haplorchis taichui*, *H. pumilio* and *Stellantchasmus falcatus*, and to evaluate potential detection in fecal specimens, and identification and differentiation of cercarial and metacercarial stages.

**Methods:**

Primers targeting the partial 28S large subunit ribosomal RNA gene were designed and about 46–47 nucleotides were selected as the target region for species identification by a PCR assay coupled with a pyrosequencing technique.

**Results:**

The nucleotide variations at 24 positions, which is sufficient for the identification of the five species of FBT were selected. The method could identify *O. viverrini* and *C. sinensis* eggs in feces, cercarial and metacercarial stages of *O. viverrini*, and metacercarial stage of *H. pumilio* and *H. taichui*. The detection limit was as little as a single *O. viverrini* or *C. sinensis* egg artificially inoculated in 100 mg of non-infected fecal sample (equivalent to 10 eggs per gram), indicating highly sensitivity. The method was found to be superior to the traditional microscopy method and was more rapid than Sanger DNA sequencing.

**Conclusions:**

DNA pyrosequencing-based identification is a valuable tool for differentiating *O. viverrini* and other *Opisthorchis*-like eggs, and can be applied to epidemiological studies and for molecular taxonomic investigation of FBT in endemic areas.

## Background

Human fishborne trematode (FBT) infections caused by liver and intestinal trematodes are especially problematic in Asian countries including Vietnam, Lao PDR, Cambodia and Thailand, for example, *Opisthorchis viverrini*, *Clonorchis sinensis, Haplorchis taichui, Haplorchis pumilio*, *Stellantchasmus falcatus*[1]. According to the World Health and the Food and Agriculture Organization of the United Nations, more than 40 million people were infected with FBT in 2002 [2]. *Opisthorchis viverrini* has been reported frequently in several countries such as Thailand, Lao PDR, South Vietnam and Cambodia [3]. *Clonorchis sinensis*, which is endemic in the People’s Republic of China, Korea, Taiwan and North Vietnam [4-6] has been reported in Thailand in one study [7]. More than 200 million people are at risk of contracting clonorchiasis, whereas 15–20 million are infected and 1.5–2 million present symptoms and complications [6]. Infection by *O. viverrini* has a major public health impact with chronic infections associated with the development cancer of the bile duct (cholangiocarcinoma) and the liver (hepatocarcinoma) in humans [8]. Since endemic areas of *C. sinensis* and *O. viverrini* are closely next to each other [1,5] with the report of co-endemic areas in Thailand [7], and since the number of travelers visiting endemic areas of those parasites has been expanding, both flukes may overlap in Southeast Asia.

The heterophyids *H. taichui* and less frequently *H. pumilio* are the most common minute intestinal flukes. *Haplorchis taichui*, *H. pumilio* as well as *S. falcatus* are prevalent and cause infections frequently in Thailand [9] and Vietnam [10]. Mixed infections of *O. viverrini* with other minute intestinal flukes of the Heterophyidae and Lecithodendriidae families have also been reported [11-16].

Diagnosis of FBT infection in humans is usually done by microscopic observation of parasite eggs in feces. However, it is difficult to differentiate *O. viverrini* and *C. sinensis* eggs as well as to discriminate opisthorchiid eggs from lecithodendriid and heterophyid eggs i.e., *H. taichui*, *H. pumilio*, *S. falcatus* and *Centrocestus caninus* because of their morphological similarity [17]. To overcome the pitfalls of traditional microscopic methods, various sensitive and specific molecular methods have been developed to discriminate between eggs of different species of FBT. However, the rapid, concurrent and high throughput identification of five species of FBT, *O. viverrini*, *C. sinensis*, *H. taichui*, *H. pumilio* and *S. falcatus* is still lacking.

Recently, DNA pyrosequencing of the PCR amplicons, the direct sequencing by the synthesis of short nucleotide fragments has been successfully applied in a variety of cases, including genotyping and species level identification of protozoan parasites [18-21], trematodes [22] and nematodes [23].

In the present study, we report the molecular identification of life cycle stages, of *O. viverrini* and *C. sinensis*, *H. taichui*, *H. pumilio* and *S. falcatus* by using the PCR assay and a high-throughput sequence analysis, pyrosequencing technique of the amplicons based on hypervariable regions within 28S rRNA genes.

## Methods

### Parasite and sample collections

Adult worms of *O. viverrini* (Khon Kaen strain, Northeast Thailand) and *C. sinensis* (Thai Binh strain, Vietnam; the generous gift from Institute of Ecology and Biological Resources, Vietnam Academy of Science and Technology, Hanoi, Vietnam) were obtained from experimentally infected hamsters and naturally infected cats, respectively, and used for genomic DNA extraction of positive control DNAs. Metacercariae of *H. taichui*, *H. pumilio* and *Stellantchasmus falcatus* were collected from naturally infected cyprinid fishes from fresh water reservoir after pepsin-HCl digestion. Human stool specimens infected with *O. viverrini* were collected from leftover specimens from patients who visited Srinagarind Hospital, Faculty of Medicine, Khon Kaen University, Khon Kaen, Thailand, and cat stool specimens infected with *C. sinensis* were obtained from Thai Binh Province, Vietnam. Cercariae of *O. viverrini* were obtained from experimentally infected *Bithynia siamensis goniomphalos* snails by light shedding technique. All of cercariae, metacercaria, eggs and adults of each specimen were morphological identified by microscopy and PCR/Sanger DNA sequencing. This study was approved by the Khon Kaen University Ethics Committee for Human Research (reference number HE541243).

### Primer design

**Figure 1:**
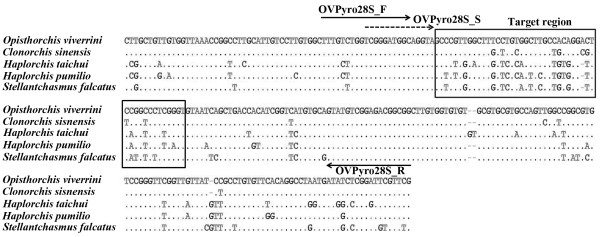
**Multiple Alignment of the 28 large subunit of ribosomal RNA from 5 fishborne trematodes.** Alignment of (28S rRNA) genes of *Opisthorchis viverrini* (HM004188), *Clonorchis sinensis* (JF823989), *Haplorchis taichui* (HM004187), *H. pumilio* (HM004191), and *Stellantchasmus falcatus* (KF241630) available in GenBank were selected to find a suitable region for discrimination these five species. The solid arrows indicate the position of OVPyro28S_F (forward primer) and biotinylated OVPyro28S_R (reverse primer) for template amplification. A dash arrow indicates OVPyro28S_S (sequencing primer), and a rectangular box shows the position of the 47 nucleotides as the target region used for species level identification. Dots indicate identical nucleotides between lines. Dash indicates gap between nucleotides.

The large subunit of ribosomal RNA (28S rRNA) genes of *O. viverrini* (GenBank:HM004188), *C. sinensis* (GenBank:JF823989), *H. taichui* (GenBank:HM004187), *H. pumilio* (GenBank:HM004191), and *S. falcatus* (GenBank:KF241630) available in GenBank were selected to find suitable regions for discrimination of these five species. After alignment of the 28S rRNA genes of those 5 species, we selected about 46–47 bp fragments in 28S rRNA genes as the suitable target region used for identification and differential detection by pyrosequencing. At this region, 24 nucleotide variations within 47 nucleotides are assumed to be sufficient for the identification of five parasites. Therefore, the conserved PCR primers (OVPyro28S_F; 5′-TTTGTCTGGTCGGGATGGC-3′ and biotinylated OVPyro28S_R; biotin-5′-CCGCCGTCTCCGACATAC-3′) and sequencing primer (OVPyro28S_S; 5′-TCGGGATGGCAGGTA-3′) were designed by using pyrosequencing assay design software (PyroMark Q96 ID software version 2.0; Biotage, Uppsala, Sweden) and used for PCR and pyrosequencing (Figure 1).

### DNA extraction and positive control plasmid preparations

Genomic DNA of all stages of parasites, 0.25 g of crushed snails tissue and grinded fish tissue were extracted using a NucleoSpin Tissue Kit (Macherey-Nagel GmbH & Co., Duren, Germany) according to the manufacturer’s protocols. Genomic DNA was eluted in 50 μl of distilled water. Two hundred milligrams of frozen feces was homogenized and extracted using the QIAamp DNA Stool Mini Kit (Qiagen, Hilden, Germany) according to the manufacturer’s protocol. DNA extracted from feces was eluted in 100 μl of distilled water.

Positive control plasmids containing 119 bp PCR products of *O. viverrini* and *C. sinensis*, and 118 bp PCR products of *H. taichui*, *H. pumilio* and *S. falcatus* 28S rRNA genes were constructed by cloning these PCR products into the pGEM®-T Easy vector (Promega, WI) according to the manufacturer’s instructions. The PCR products were obtained from conventional PCRs using the OVPyro28S_F and OVPyro28S_R primers. The recombinant plasmids were propagated in *Escherichia coli* JM109. For confirmation and validation of sequence information, the nucleotide sequences of the inserted genes were sequenced in both directions.

### Polymerase chain reaction (PCR) and pyrosequencing assays

Conventional PCR was performed with OVPyro28S_F and biotinylated OVPyro28S_R primers which were used for amplification region of 28S rRNA gene. The PCR reaction was carried out in a 25 μl final reaction volume, which composed of 1× PCR buffer (Invitrogen, Carlsbad, CA) with 0.2 mM each of dNTPs, 1.5 mM MgSO_4_, 0.2 μM each of OVPyro28S_F primer and biotinylated OVPyro28S_R primer, 0.625 U of Platinum *Taq* DNA polymerase high fidelity (Invitrogen) and 2 μl of the DNA sample in a GeneAmp PCR system 9700 thermal cycler (Applied Biosystems, Singapore). The amplification procedure was as follows: 5 minutes at 94°C for initial denaturation followed by 35 cycles of denaturation for 30 seconds at 94°C, annealing for 30 seconds at 60°C, and extension for 30 seconds at 72°C, followed by a final extension for 7 minutes at 72°C. Two microliters of the PCR products was used to detect and verify the molecular size of PCR products on a 1% agarose gel. The specific amplicon products that contained biotinylated primer were then prepared before performing pyrosequencing. To check the reliability of pyrosequencing data, all PCR products of each sample that was amplified with OVPyro28S_F and non-biotinylated OVPyro28S_R primers similarly with the above condition, was then subjected to sequencing by the Sanger method and compared with the pyrosequencing data. The pyrosequencing assays were performed by using the PyroMark™ Q96 ID instrument (Biotage, Uppsala, Sweden) according to the manufacturer’s protocols. The procedure was similar as previously described [20] with the exception that 0.4 μM OVPyro28S_S specific sequencing primer was used before performing pyrosequencing reaction.

### Analytical sensitivity and specificity

To determine sensitivity of egg detection in feces, 2 μl of DNA extracted from 100 mg of non-infected hamster and cat feces obtained from the Northeast Laboratory Animal Center, Khon Kaen University which were artificial mixed with 1, 2, 5, 10 and 30 eggs of *O. viverrini* and *C. sinensis* were also examined, respectively. The spiking experiments were repeated in triplicate. For the evaluation of analytical specificity, 1 μl (3 ng) of control genomic DNAs from other organisms were analyzed. The panel of other organisms was *Necator americanus*, *Strongyloides stercoralis*, *Ascaris lumbricoides*, *Trichuris trichiura*, *Capillaria philippinensis*, *Taenia* spp., *Schistosoma mekongi*, *S. japonicum*, *Echinostoma malayanum*, *Fasciola gigantica*, *Paragonimus heterotremus*, *Isospora belli, Trichostrongylus* spp. and *Giardia lamblia*. Genomic DNAs of human leukocytes as well as DNA from non-infected fishes and snails tissues, and DNA from non-infected hamster and cat feces were also included. To compare efficacies of microscopic examination, Sanger DNA sequencing and pyrosequencing assays, identification and diagnosis of *O. viverrini* infected-human (n = 5) and *C. sinensis* infected-cat (n = 5) feces, individual opisthorchiid-like cercaria (n = 4) from infected snails and individual opisthorchiid-like (n = 3) or *Haplorchis*-like (n = 3) metacercaria from infected fishes were performed.

## Results

### Multiple alignment of 28S rRNA sequences and pyrosequencing assays

**Figure 2:**
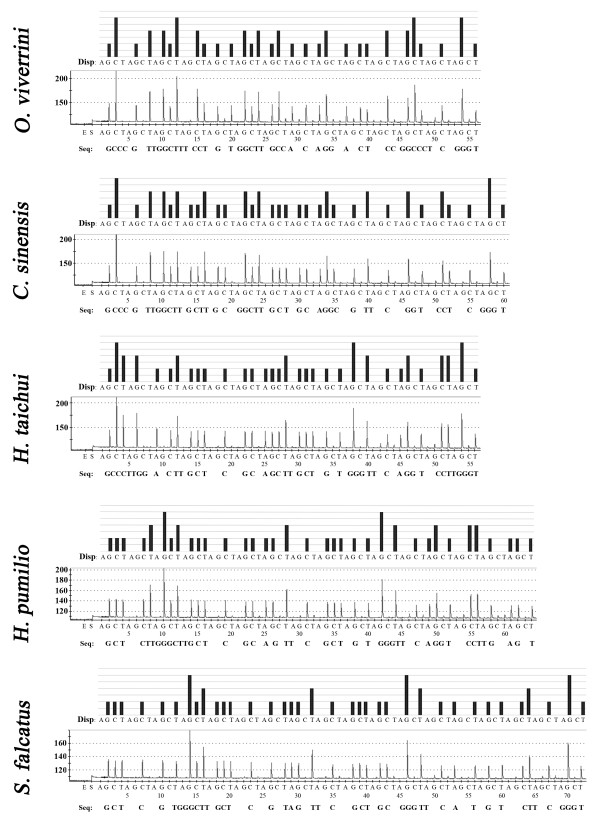
**Pyrosequencing data of 5 fishborne trematodes.** Theoretical pyrogram patterns (top of each panel) and a representative raw data (bottom of each panel) of *Opisthorchis viverrini*, *Clonorchis sinensis*, *Haplorchis taichui*, *H. pumilio*, and *Stellantchasmus falcatus* from pyrosequencing. Pyrosequencing based on the large subunit of ribosomal RNA genes was performed by addition of enzyme (E), substrate (S), and four different nucleotides. The letters under the black bars show the dispensation (Disp:) order. The actual sequence detected by pyrosequencing is indicated below the panels after “Seq:”. Y-axis represents the level of fluorescence emitted by the incorporation of a nucleotide base and X-axis represents the total number of bases added at that point in time; A, G, C, T, nucleotide bases.

**Figure 3:**
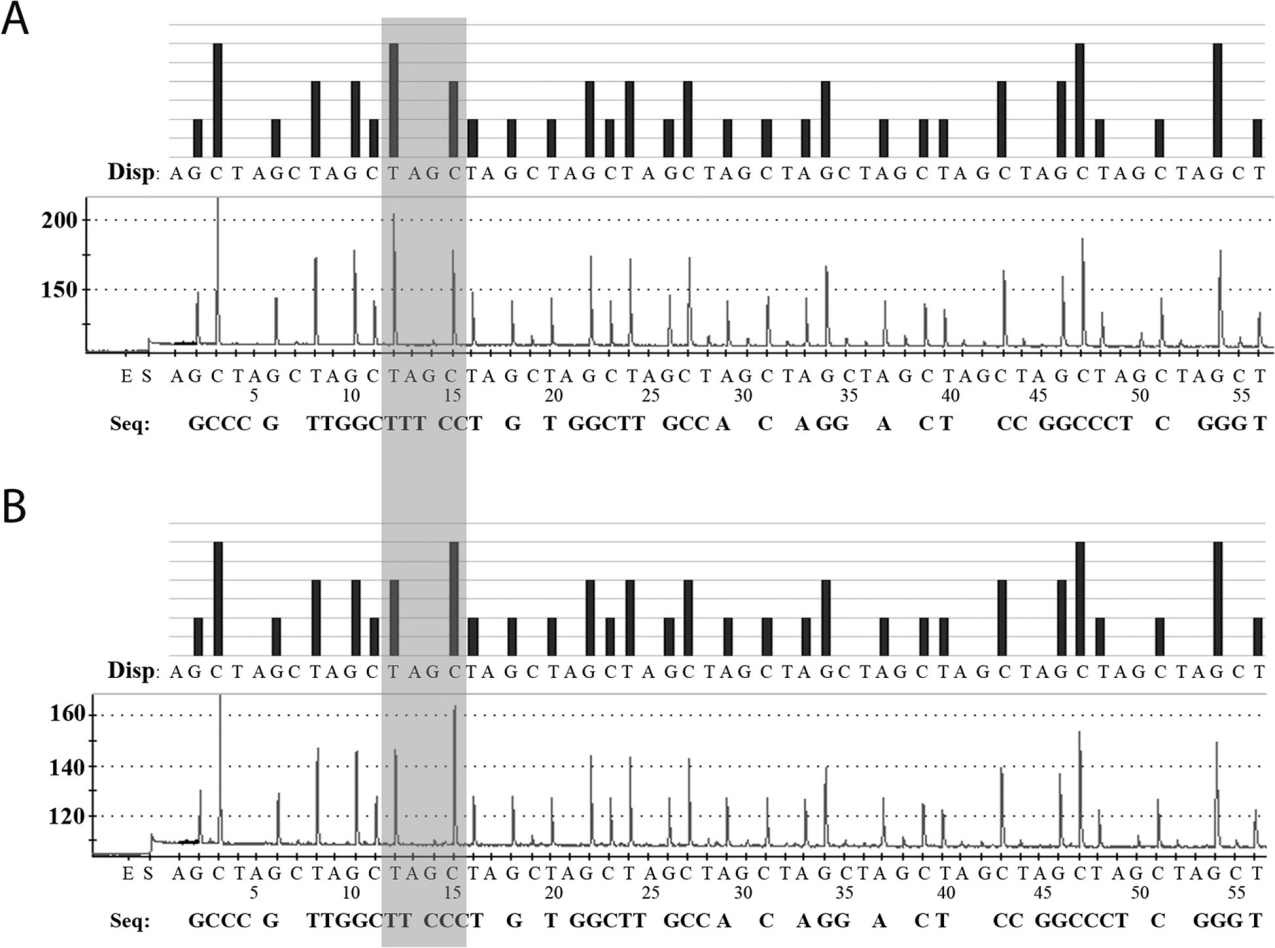
**Single nucleotide polymorphisms of *Opisthorchis viverrini*.** Theoretical pyrogram patterns (top of each panel) and a representative raw data (bottom of each panel) of *Opisthorchis viverrini* from pyrosequencing. The areas show the pyrograms for the species differentiation in this cluster. Single nucleotide polymorphisms, exhibited in the light gray in the program were analyzed to discriminate thymine **(A)** and cytosine **(B)** of *O. viverrini*.

Multiple alignment of the 28S rRNA genes from *O. viverrini* (GenBank:HM004188), *C. sinensis* (GenBank:JF823989), *H. taichui* (GenBank:HM004187), *H. pumilio* (GenBank:HM004191), and *S. falcatus* (GenBank:KF241630) available in GenBank showed that 47 nucleotides (for *O. viverrini* and *C. sinensis*) and 46 nucleotides (for *H. taichui*, *H. pumilio*, and *S. falcatus*) after the 3′ end of sequencing primer as the target region was sufficient for identification of the five target FBT by pyrosequencing technique (Figure 1 and Table 1). The nucleotide sequence profiles at the target regions of each flukes were demonstrated as the pyrogram results (Figure 2). Within 46–47 nucleotides, 24 positions were nucleotide variations, which was useful in classifying and discriminating the sequenced FBT species (Table 1). Among 5 species, *O. viverrini* could be discriminated from the others by seven nucleotide positions (T/C13G, C15T, C26T, A27G, A32C/-, C33G/T, and C39T) and *C. sinensis* distinct from the others by three nucleotide positions (C32A/-, G33C/T, and T35C) (Table 1 and Figure 2). By using this region as the marker, *H. taichui* and *H. pumilio* separated from *O. viverrini*, *C. sinensis* and *S. falcatus* by 3 nucleotide positions (T5G, T28C and T43C), while *H. taichui* and *H. pumilio* can be differentiated each other by using 5 nucleotide positions (C3T, A9G, C21T, T23C, and G45A). Similarly, *S. falcatus* have two unique nucleotides at the position number 37 (T) and 41 (T), which were used for identification from the others. All samples of *O. viverrini*, *C. sinensis*, *H. taichui* and *H. pumilio* and *S. falcatus* included genomic DNAs and positive control plasmids exhibited the same sequence except the T → C substitution at the position no. 13 of *O. viverrini* was found (Figure 3). The sequences of five trematodes by pyrosequencing showed the same sequences with Sanger sequencing results (Additional file 1: Figure S1).

**Table 1:** **Regions of the 28S large subunit of ribosomal RNA genes (28S rRNA) and nucleotide patterns used to differentiate *Opisthorchis viverrini*, *Clonorchis sinensis*, *Haplorchis taichui*, *H. pumilio*, and *Stellantchasmus falcatus *** **by the pyrosequencing technique**

**Types (Accession No.)**	**Nucleotide positions after the 3′ end of sequencing primer**																							
	**3**	**5**	**7**	**9**	**13**	**15**	**16**	**18**	**19**	**21**	**23**	**26**	**27**	**28**	**29**	**32**	**33**	**35**	**36**	**37**	**39**	**41**	**43**	**45**
***O. viverrini*** **(HM004188)**	C	G	T	G	T/C^a^	C^a^	T	T	G	C	T	C^a^	A^a^	C	A	A^a^	C^a^	C	C	G	C^a^	C	C	G
***C. sinensis*** **(JF823989)**	C	G	T	G	G	T	T	C	G	C	T	T	G	C	A	C^b^	G^b^	T^b^	C	G	T	C	C	G
***H. taichui*** **(HM004187)**	C^d^	T^C^	G	A^d^	G	T	C	C	A	C^d^	T^d^	T	G	T^C^	G	-	T	C	A	G	T	C	T^C^	G^d^
***H. pumilio*** **(HM004191)**	T^d^	T^C^	G	G^d^	G	T	C	C	A	T^d^	C^d^	T	G	T^C^	G	-	T	C	A	G	T	C	T^C^	A^d^
***S. falcatus*** **(KF241630)**	T	G	G	G	G	T	C	T	A	T	C	T	G	C	G	-	T	C	A	T^e^	T	T^e^	C	G

^a^Nucleotide for identification of *O. viverrini*; ^b^Nucleotide for identification of *C. sinensis*; ^c^Nucleotide for identification of *H. taichui* and *H. pumilio*; ^d^Nucleotide positions used for discrimination between *H. taichui* and *H. pumilio*; ^e^Nucleotide for identification of *S. falcatus*.

### Specificity and sensitivity analysis

**Figure 4:**
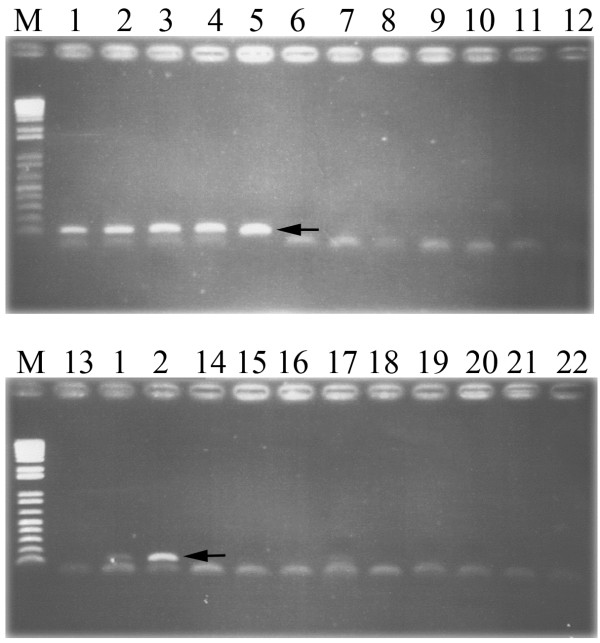
**The amplification products from the analytical specificity testing by conventional PCR.** Specificity analysis of amplification of approximate 118–119 bp of 28S rRNA product by OVPyro28S_F and OVPyro28S_R primers with *Opisthorchis viverrini* (1), *Clonorchis sinensis* (2), *Haplorchis taichui* (3), *H. pumilio* (4), *Stellantchasmus falcatus* (5), *Ascaris lumbricoides* (6), *Strongyloides stercoralis* (7), *Necator americanus* (8), *Trichuris trichiura* (9), *Capillaria philippinensis* (10), *Taenia* spp. (11), *Schistosoma mekongi* (12), *S. japonicum* (13), *Echinostoma malayanum* (14), *Fasciola gigantica* (15), *Paragonimus heterotremus* (16), *Isospora belli* (17), *Trichostrongylus* spp. (18), *Giardia lamblia* (19), genomic DNAs of human leukocytes (20), DNA from non-infected fishes (21) and snails tissues (22). M = 100 bp DNA ladder. Arrows indicate approximate 118–119 bp of amplified PCR product.

The specificity of the PCR primer set was analyzed and the results showed that no amplification product was detected with DNAs of other parasites (see Methods) or genomic DNAs from human leukocytes or uninfected cat and hamster feces or snail and fish tissues (Figure 4). Furthermore, pyrosequencing result of other organisms and negative control showed no pyrogram (Additional file 2: Figure S2). The method could detect as little as either a single egg of *O. viverrini* or a single egg of *C. sinensis* artificially inoculated in 100 mg of non-infected hamster and cat feces, respectively.

### Detection efficacy of pyrosequencing compared with microscopic examination and Sanger sequencing

For evaluation of the detection efficacy of the applications, DNAs extracted from the fecal specimen of five human opisthorchiasis patients and two opisthorchiid-like infected metacercariae from infected fishes as well as four unidentified cercariae from infected snails by microscopic examination were used. The results showed that the samples, which present the *O. viverrini* sequences pattern were similar to the *O. viverrini* positive control plasmid (Table 2). Moreover, DNAs extracted from five *C. sinensis* infected cat feces which have been previously identified by microscopic examination and Sanger DNA sequencing, could be confirmed as the *C. sinensis* with the same sequence by pyrosequencing (Table 2). Additionally, three *Haplorchis*-like metacercariae which was previously identified by microscopic examination similarly showed the Sanger DNA sequencing and pyrosequencing results with two *H. pumilio* and one *H. taichui* (Table 2). Interestingly, one opisthorchiid-like metacercaria which was first identified by microscopical examination exhibited the *H. pumilio* pyrogram (Table 2).

**Table 2:** **Comparative efficacies of microscopy, Sanger DNA sequencing and pyrosequencing assays in the identification and detection of *Opisthorchis viverrini*, *Clonorchis sinensis*, *Haplorchis taichui *** **and *H. pumilio *** **in various samples**

	**Sample size**	**Identification by**		
**Samples**		**Microscopic examination**	**Sanger DNA sequencing**	**Pyrosequencing**
Human Stool	5	*O. viverrini*	*O. viverrini*	*O. viverrini*
Cat stool	5	*C. sinensis*	*C. sinensis*	*C. sinensis*
Cercaria from snail	4	Opisthorchiid-like	*O. viverrini*	*O. viverrini*
Metacercaria from fish	2	Opisthorchiid-like	*O. viverrini*	*O. viverrini*
Metacercaria from fish	4	Opisthorchiid-like (n = 1)	*H. pumilio*	*H. pumilio*
		*Haplorchi*s-like (n = 3)	*H. pumilio* (n = 2)	*H. pumilio* (n = 2)
			*H. taichui* (n = 1)	*H. taichui* (n = 1)

## Discussion

The FBT, including liver and intestinal flukes, are recognized as an important group of emerging and re-emerging human pathogens especially to populations living in low- and middle-income countries [24]. Two species of liver flukes, *O. viverrini* and *C. sinensis*, and more than 50 species of intestinal flukes, including *Haplorchis* spp., *S. falcatus*, and *Centrocestus* spp. are important representative examples of the FBT in Southeast Asia [24]. Differential detection of the FBT eggs in fecal specimens is difficult and frequently results in misidentification due to similar morphology [17] especially in co-infection areas [12,25,26].

Many molecular techniques have been developed for the detection of a range of FBT, i.e., multiplex PCR and real-time PCR for identification of *C. sinensis* and *O. viverrini*[27,28], random amplified polymorphic DNA-PCR method for differential detection of *O. viverrini* and *H. taichui*[29], PCR targeting ITS regions for *O. viverrini*, *C. sinensis*, *H. pumilio* and *H. taichui*[30], PCR-restriction fragment length polymorphism (RFLP) for detection of *O. viverrini*, *C. sinensis*, and *H. taichui*[31] and for *H. taichui*, *H. pumilio*, *H. yokogawai*, *Procerovum varium*, *S. falcatus*, and *Centrocestus formosanus*[32]. Our study could identify life cycle stages of *O. viverrini*, eggs in infected feces, metacercariae from infected fishes, and cercarial stages from infected snails; *C. sinensis* eggs in infected feces as well as *H. pumilio*, *H. taichui* and *S. falcatus* metacercariae from infected fishes at the species level by a pyrosequencing approach. Because this primer set was designed based on the highly conserved regions of the 28S rRNA gene from 5 FBT examined herein, it could have major applications in the identification and differentiation of other FBT.

In the present study, our developed pyrosequencing technique is a rapid and high throughput method, which can obtain results from 96 samples within 1 hour after PCR amplification. The reasonable cost estimate for pyrosequencing for a 96 sample analysis was $220 ($4.58 per sample). This was an approximate estimation and would vary depending upon the commercial source of reagents used. To check the reliability of pyrosequencing data, PCR amplicons were sequenced by the Sanger method and compared with the pyrosequencing data. Both sets of sequence data were completely identical with each other at the 46–47 nucleotides of target region. Furthermore, the primer set used in this present study showed specificity with the 5 FBT and no amplification product was found with control genomic DNAs.

There are several advantages of the pyrosequencing technique compared with Sanger sequencing; (i) pyrosequencing determines the exact sequence thereby providing the same accuracy as conventional sequencing methods, (ii) the technique dispenses with the need for labeled nucleotides, labeled primers and electrophoresis, (iii) large number of samples can be analysed in a short time, the work included analyses of test samples at the specified rate of 96 samples within 1 hour and (iv) the reagent costs are considerably lower for sequencing short stretches of DNA compared to currently available methods [33]. These are the reasons why this method could be the best-alternative way for molecular identification. However, there are some limitations; (i) pyrosequencing is easily capable of detecting PCR fragments that are 25–50 bp in length while longer fragments may pose a problem and (ii) the target sequence contained up to four homopolymers was limited for software interpretation. However, this problem can be clarified by manual interpretation.

Our pyrosequencing assay showed high sensitivity with the limit of detection of a single egg of *O. viverrini* and *C. sinensis* in feces, equivalent to 10 EPG. This result is quite similar with previous reports for the egg detection limits of either *O. viverrini* or *C. sinensis* single egg by PCR in fecal samples [31,34] as well as can be detected with ranges of 5–10 EPG in fecal samples by a single plex Taqman probe-based real-time PCR assay for *C. sinensis* eggs [35,36], by a single plex FRET-probe-based real-time PCR assay for *O. viverrini* eggs [37] and by a duplex real-time FRET PCR assay for *C. sinensis* eggs [28]. Variations in the detection limits noted in various reports were possibly due to inhibitors remaining in the fecal samples. Interestingly, we found nucleotide variation within *O. viverrini* at the position no. 13 (T → C substitution), indicating that this method can be implied for opisthorchiid flukes genotyping, similar to a previous report of the genotyping of *Blastocystis*[19].

## Conclusions

In conclusion, the pyrosequencing technique can be a practical alternative method used in a high-throughput format for molecular detection of 5 species of FBT in Southeast Asia. This rapid, accurate and cost-effective assay can be applied to identify several life cycle stages including eggs, cercariae, and metacercariae. However, this developed technique should be evaluated using larger sample sizes and more varied isolated samples or different parasite strains before practical use in clinical and field samples. Therefore, the method can have important implications for molecular genotyping and epidemiological studies of all five FBT in endemic countries.

## Competing interests

The authors declare that they have no competing interests.

## Authors’ contributions

CT, PMI, VL and WM took part in the conception, planned and designed the protocols. CT, TT, OS, PJ and LS performed laboratory work and samples collection. CT performed primer design, laboratory work and prepared the manuscript. PMI and WM reviewed the drafts of the manuscript for important intellectual content. All authors have seen and approved the final version of the manuscript.

## Supplementary Material

Additional file 1: Figure S1 Sanger sequencing results. Nucleotide Sequences of 28S ribosomal RNA of *Opisthorchis viverrini*, *Clonorchis sinensis*, *Haplorchis taichui*, *H. pumilio*, and *Stellantchasmus falcatus* by Sanger method. Highlighted nucleotides indicate sequencing primer position. Click here for file

Additional file 2: Figure S2 Pyrosequencing results of other organisms and negative control. Representative pyrosequencing results of other organisms such as *Necator americanus* (a), *Strongyloides stercoralis* (b), *Ascaris lumbricoides* (c), *Trichuris trichiura* (d) and negative control (e) showed no pyrogram. Click here for file
