# Supplementary figures and images for: Development of a PCR assay and pyrosequencing for identification of important human fish-borne trematodes and its potential use for detection in fecal specimens
Source: Parasit Vectors. 2014 Mar 3;7:88. doi: 10.1186/1756-3305-7-88 (PMC3943809; doi:10.1186/1756-3305-7-88)

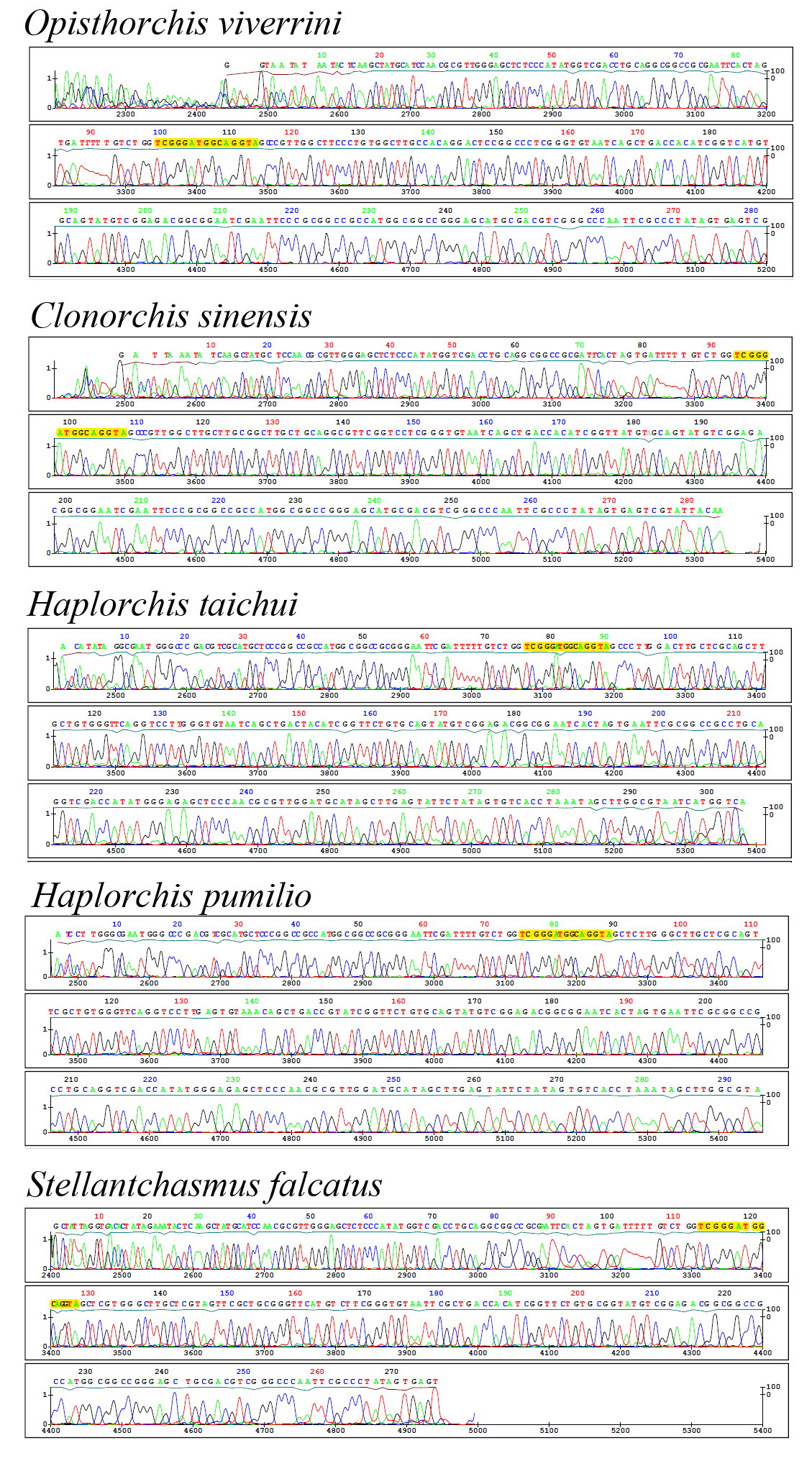

Supplement: Additional file 1: Figure S1 — Sanger sequencing results. Nucleotide Sequences of 28S ribosomal RNA of Opisthorchis viverrini, Clonorchis sinensis, Haplorchis taichui, H. pumilio, and Stellantchasmus falcatus by Sanger method. Highlighted nucleotides indicate sequencing primer position. [file 1756-3305-7-88-S1.tiff]

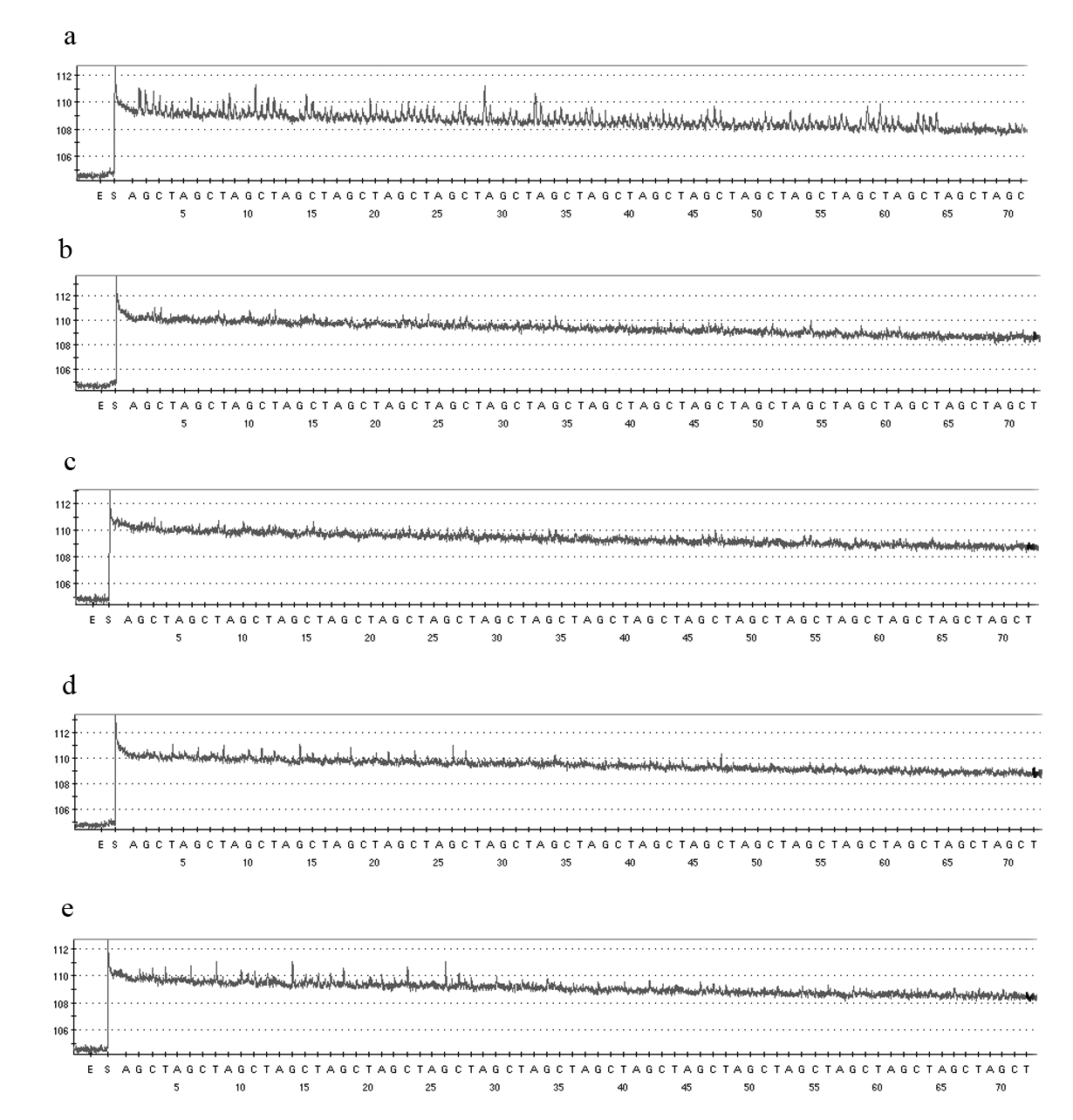

Supplement: Additional file 2: Figure S2 — Pyrosequencing results of other organisms and negative control. Representative pyrosequencing results of other organisms such as Necator americanus (a), Strongyloides stercoralis (b), Ascaris lumbricoides (c), Trichuris trichiura (d) and negative control (e) showed no pyrogram. [file 1756-3305-7-88-S2.tiff]
